# A Content Analysis of Osteopaths’ Attitudes for a More Inclusive Clinical Practice towards Transgender People

**DOI:** 10.3390/healthcare10030562

**Published:** 2022-03-17

**Authors:** Irene Baldin, Jorge E. Esteves, Marco Tramontano, Mia Macdonald, Francesca Baroni, Christian Lunghi

**Affiliations:** 1 Malta ICOM Education, GZR 1071 Santa Venera, Malta; irene.baldin@gmail.com (I.B.); osteojorge@gmail.com (J.E.E.); 2 Foundation COME Collaboration, 65100 Pescara, Italy; fbaroni@comecollaboration.org (F.B.); clunghi@comecollaboration.org (C.L.); 3 Fondazione Santa Lucia IRCCS, 00179 Rome, Italy; 4 Centre Pour l’Etude, la Recherche et la Diffusion Osteopathiques, 00199 Rome, Italy; 5 Department of Health, Swiss Distance University of Applied Sciences (FFHS), 8105 Regensdorf, Switzerland; mia.macdonald@ffhs.ch; 6 Unit of Research in Mobility & Musculoskeletal Care (URM), School of Health Sciences Fribourg, University of Applied Sciences and Arts Western Switzerland (HES-SO), 1700 Fribourg, Switzerland

**Keywords:** transgender, microaggressions, cisnormativity, osteopathic medicine, inclusivity

## Abstract

Objectives. The aim of this qualitative study was to explore the attitudes, beliefs, and preferences of Italian osteopaths regarding the management of transgender patients through a content analysis of emergent data from semi-structured interviews. Methods. This study was a content analysis based on the Standards for Reporting Qualitative Research guidelines. Purposive sampling of 10 Italian osteopaths was applied. Data were collected through semi-structured interviews, from March to April 2021, and subsequently transcribed verbatim with the content analysis carried out as an iterative process. Results. One participant was excluded during the first interview due to them being unsuitable for this study. Data saturation was reached after two interviews with the remaining nine participants. Data analysis revealed four main themes: microaggressions, acceptance and non-judgement, person-centered treatment, and education implementation. Conclusions. This study presents cisgender Italian osteopaths’ attitudes in the care of transgender people, revealing the desire to embrace and apply osteopathic tenets regardless of the patient’s gender identity.

## 1. Introduction

Effective communication is fundamental in establishing a proactive relationship between the patient and the practitioner, and provides positive messages and avoids misunderstandings due to technical jargon [1,2,3]. Although there are specific communication approaches depending on health conditions and the type of population concerned [2,3], there are a lack of approaches within healthcare settings regarding the LGBTQIA+ (lesbian, gay, bisexual, transgender, queer/questioning, intersex, asexual/aromantic/agender and others) community (Appendix A. Table A1), who are often discriminated against in the form of microaggressions [4]. Microaggressions are behaviors, affirmations, and environmental messages that subconsciously convey hostile beliefs about ethnicity, gender, sexual orientation, and religion [4]. The transgender population is even more exposed to microaggressions during healthcare sessions, leading to a worse quality of life compared with LGB counterparts [5,6,7,8,9,10,11]. Intersex people (i.e., those born with biological sex characteristics not conforming with the male–female gender binarism, or later develop them in puberty) suffer from similar circumstances [12]. Discrimination towards transgender people is related to cisnormativity, which makes the healthcare system less accessible for this population [11,12,13,14]. Microaggressions commonly occur unconsciously [4,15], and may limit the shared decision-making approach and undermine clinical outcomes [16]. Microaggressions can ruin trust in the relationships between patients and practitioners, which is fundamental for therapeutic success [17]. This could even lead to the avoidance of healthcare practitioners by transgender people [4].

A review published in the Annals of Internal Medicine in 2019 [18] reported health disparities in transgender people, such as the greater prevalence of certain types of cancer, substance misuse, mental health disorders, infections, and chronic diseases. LGBTQIA+ individuals have higher rates of anxiety and depression, and are at increased risk for specific medical conditions such as obesity, breast cancer, and human immunodeficiency virus [19]. These conditions are exacerbated by barriers to accessing appropriate and culturally competent care [19]. As a result, practitioners must be aware of the specific medical conditions that affect this demographic [18]. Clinical research efforts, together with enhanced clinician education and training, hold the possibility of improving overall health and quality of life for older LGBT people [18,19].

A recently published cross-sectional, web-based study looked at potential differences in gender identity milestones, minority stress, and mental health among three generations of Italian transgender and gender non-conforming people, using the minority stress theory and a life-course perspective [20]. Data show that changes in social context have a minimal impact on transgender and gender non-conforming people’s stress processes and health. This should encourage Italian academics, policymakers, and activists to continue to promote transgender and gender non-conforming people’s equality by implementing inclusive practices in the primary socialization environments where they live and communicating equality messages to the entire society.

The lack of education, up to informational erasure (i.e., the lack of LGBTQIA+ information within education programs), concerning specific needs of transgender people does not make healthcare practitioners competent in the management of transgender patients, hence resulting in microaggressions [11]. Several studies have demonstrated that general healthcare practitioners lack cultural competence from the perspective of transgender patients [6,8,21]. This lack is evident in the context of osteopathic care. Indeed, within the osteopathic community, research findings have highlighted the lack of cultural and communicative competencies and the need to include more diversity efforts related to the LGBTQIA+ population [22]. Nevertheless, osteopathy is a patient-centered, hands-on, whole-body intervention to enhance self-regulation [23]. OMT person-centered interventions mainly focus on somatic dysfunctions [24,25,26], which can be palpable in different body regions remote from the symptomatic area [27]. Principles of osteopathic care have recently been proposed as an intersectional framework for approaching patients’ sexual orientation and disclosure of their gender identity [21,28]. A qualitative review published in 2021 [28] explored how the Four Tenets of Osteopathic Medicine are applied when a patient self-discloses their sexual orientation and gender identity to their healthcare provider, popularly known as coming out.

Intersectionality and coming out are discussed in the light of Tenet One: the body is a unit; a person is a unit of body, mind, and spirit. Tenet Two states that the body can self-regulate, self-heal, and maintain health, which promotes a better understanding of how coming out promotes homeostasis and self-healing. Tenet Three claims that structure and function are reciprocally interrelated. It looks at how structure and function can be understood on a human level and how society affects how people come out. Tenet Four argues that osteopathic care is based upon an understanding of the basic principles of body unity, self-regulation, and the interrelationship of structure and function. It explains the resources that are accessible to help with the changes described earlier. The provider will be better able to comprehend what “coming out” means on a personal and societal level, as well as the ramifications for their patient’s health if the Four Tenets are followed. Even though the osteopathic tenets can be applied to transgender people, it is still important to ascertain to what extent this is fully realized with transgender people and whether improvements to the current approaches are needed. We hypothesize that interviewing health practitioners on this topic could help understand the limits and difficulties of osteopaths in managing transgender people. For these reasons, this study aimed to explore the attitudes, beliefs, and preferences of Italian osteopaths to promote a more inclusive practice for transgender people through content analysis of emergent data from semi-structured interviews.

## 2. Materials and Methods

**Figure 1:**
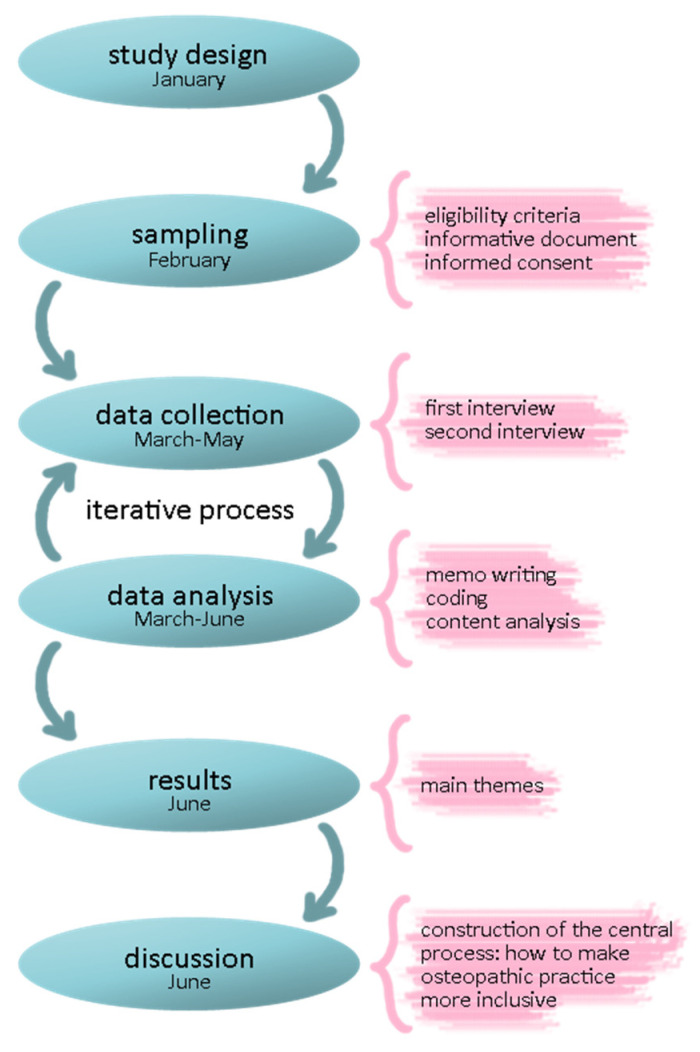
Study design and methods.

The methodology implemented in previous qualitative research conducted in the osteopathic field was followed [17], adopting a descriptive phenomenological approach [29] with content analysis [30]. Descriptive phenomenology is an effective theoretical approach for understanding subjective experience and gaining insights into people’s actions and motivations, questioning traditional knowledge, and cutting through long-held preconceptions. It can aid in creating new theories, policy changes, or response changes [29]. The authors referred to the Standards for Reporting Qualitative Research (SRQR) checklist [31] to ensure that the methods and development of the study were reliable (Figure 1). The present qualitative study was registered with protocol number 10.17605/OSF.IO/U83QV on the Open Science Framework Registry.

Two semi-structured interviews were carried out to evaluate attitudes, beliefs, and preferences. The ethical aspects, as well as the data collection and analysis, are reported in the following sections.

### 2.1. Ethics

This study was approved by the Malta ICOM (International College of Osteopathic Medicine) institutional review board (4 March 2021 n. BI000434BAFT). The study was conducted in accordance with the Declaration of Helsinki [32].

### 2.2. Study Group, Selection and Recruitment

In order to capture the variability of the phenomenon, purposive sampling was performed to screen participants with experience in treating transgender patients [33]. The number of participants was determined by data saturation and fell within the acceptable number for phenomenological studies (5–25 participants) [34].

To reach data saturation [35], 10 Italian cisgender osteopaths were recruited through the Italian Register of Osteopaths database. The recruitment document explained that participation was voluntary, without incentives for participants, and was dependent on the eligibility criteria. All interested participants received information about the project by mail.

Eligibility criteria required participants to be Italian-speaking, practicing osteopaths with at least 10,000 clinical activity hours [36], as well as having experience with at least one transgender patient. The authors followed the methodology for anonymizing the sensitive details described by Saunders et al. [37]: names, places, religious or cultural background, occupation, family relationships, and other potentially identifying information was removed. Participants’ anonymity was guaranteed by assigning an identification code (O1–O10) to each of them. The list of names and codes was only accessible to I.B. Participants could freely drop out of the study at any moment until data collection was completed.

### 2.3. Data Collection and Analysis

#### 2.3.1. Data Collection

Semi-structured interviews were used; the evolving processes of interview and observation enabled participants to acquire new insights into the phenomena of the study to subsequently influence follow-up questions or narrow the focus for observation, contributing to richer variation [38,39]. Individual semi-structured interviews [38,39] were conducted on two different occasions, each approximately 20 min long [35]. Open questions were asked to participants about their clinical practice with transgender patients. The first interview (Appendix B) focused on the relationship between patient and practitioner, the completion of the case history, and the practitioner’s attention to creating an inclusive clinic environment (gender-neutral bathrooms, etc.). After one month, the second interview was carried out and the questions (Appendix B) were generated following an iterative process to investigate new emergent themes [39,40] and addressed the practitioner’s ability, multidisciplinary approach, and university education. Data saturation was reached with the second interview, which did not bring up any new themes to be examined with other interviews. Interviews were declared completed when the fields of interest were saturated.

The strategy presented by Guest et al. [35,41] was employed to accomplish the saturation process, defining a stop if the new information collected fell below 5% (Table 1). Base size refers to the information identified in the dataset (i.e., the codes representing themes) and was used as a denominator in calculating the new information threshold [41]. The *run length* was a set of successive interviews used as a numerator while looking for the *new information threshold* [41]. We used a *base size* of 4 and a *run the length of* 2, which meant consecutive runs overlapped by one shifting to the right. The *new information threshold* was the ratio between the *base size* and *run length*, and represented data saturation, where 0% meant no new information [41].

Interviews were carried out by the principal investigator (I.B.) using a video conference platform on an arranged date and time to meet participants’ needs. No-one was present besides the interviewer and the individual participant. To maintain high-quality recordings and prevent difficulties with audio quality later in the research process, the interviewer paid attention to excessive background noise, weak batteries of the laptop computer, placement of the laptop computer, and other issues influencing the quality of recorded interviews. The ethical issues related to the interview process were considered to reduce the risk of unanticipated harm, protect the interviewee’s information, effectively inform interviewees about the nature of the study, and reduce the risk of exploitation [34]. The interviewer was an osteopath with curricular training on implementing qualitative research projects. Before proceeding with the interviews, the questions were tested with C.L., an osteopath with more than 10,000 h of clinical, educational, and research practice [36]. It was ensured that there were no relationships between the interviewer and the participants prior to this study. As written in the informed consent form, interviews were recorded and stored on a password-protected online platform.

Field notes were taken during the interviews to support the subsequent content analysis [42]. Before carrying out the analysis, each participant checked the verbatim transcriptions of their interviews and confirmed that the transcriptions represented their thoughts whilst maintaining their anonymity. Only one participant (O6) corrected the second interview syntax without altering the meaning.

#### 2.3.2. Data Content Analysis

The analysis was carried out in an inductive way, following the methodology for descriptive phenomenological qualitative studies outlined by Giorgi [29]. Codes, which are significant parts of data, were identified after thorough readings of the transcriptions supported by field notes [39,40]. Codes were grouped into abstract categories through a conceptual analysis to discover essential aspects of the examined themes and generate a thematic map [33,34]. As a result of the analysis, the codes were viewed as essences, and their relationships were clarified through an overarching theme, sub-themes, and categories. Throughout the data analysis, the researchers (I.B. and C.L.) discussed the procedure in a continuous and collaborative manner in order to arrive at a consensus.

### 2.4. Reliability 

This study used several methodological approaches recommended by Graneheim and Lundman [30] to improve the credibility of the qualitative data. Researchers typically conducted peer debriefings to identify and investigate themes and categories to increase the reliability of the data. The debriefings allowed researchers to reflect on their roles and identify any biases, preferences, and personal impacts on the analysis. Before the data analysis process, researchers shared their assumptions and prejudices. Researchers detailed the study’s methodological approaches to improve dependability and documented the procedure to facilitate replication. Moreover, the researchers confirmed that their conclusions were based on data by a sharing process to improve confirmability.

Furthermore, an audit trail method was employed to enhance dependability and confirmability: the researcher who was least involved in data collection (C.L.) oversaw the entire procedure. Finally, C.L. compared the overarching themes, motifs, and categories with the original transcriptions to improve internal consistency. Ultimately, the characteristics of the individuals were presented in order for the findings to be transferable.

## 3. Results

Ten cisgender osteopaths (four women and six men) participated in this study, although one of them (O3) was excluded from data collection because, during the first interview, they showed confusion about the meanings of “transgender” and “homosexual” and, after a brief explanation, admitted to not having had experience with transgender patients. Demographic data of the participants are reported in Appendix C (see Table A2). The analysis revealed one overarching theme, four themes, and eleven categories emerging from the interviews, which shed light on osteopaths’ attitudes and demeanor towards transgender people (Table 2). The themes are described below, along with some supporting quotations extracted verbatim. The categories in the results are intended to represent the essential concepts in each theme. Participants are identified by the code within parentheses following the verbatim quotations (O1–O10).

**Figure 2:**
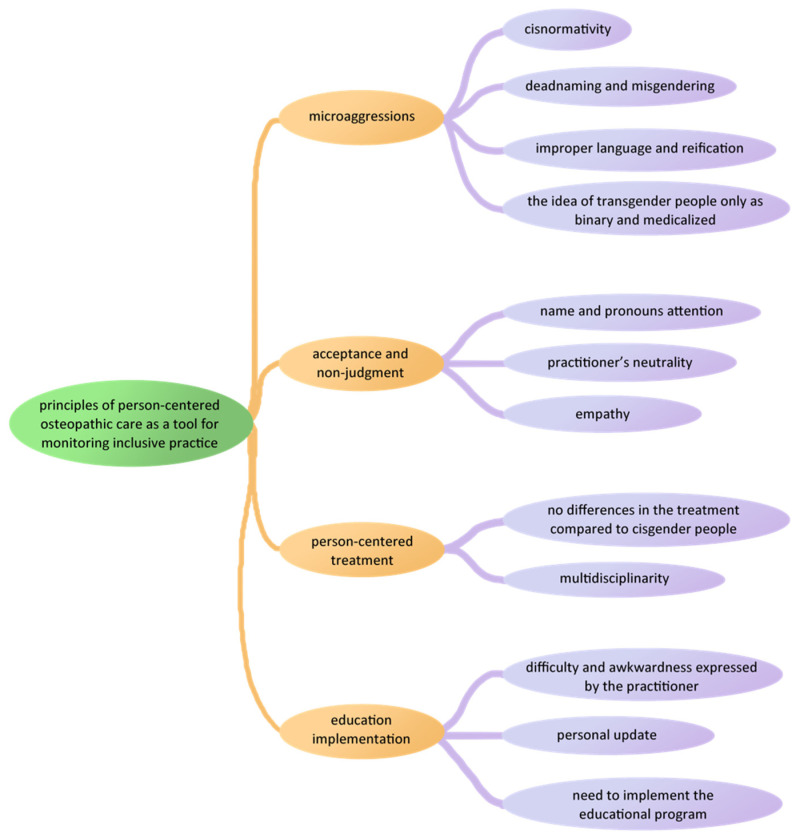
Osteopaths’ attitudes toward transgender patients: a thematic map.

The process of content analysis has been schematized in Figure 2. The findings are presented with the themes and the descriptive text, illustrated with some extracted verbatim quotations that support them. All participants’ quotes are reported in Table 3, Table 4, Table 5, Table 6, Table 7, Table 8, Table 9, Table 10, Table 11, Table 12, Table 13 and Table 14.

### 3.1. Principles of Person-Centered Osteopathic Care as a Tool for Monitoring Inclusive Practice

Osteopaths described their experience in applying osteopathic principles in a person-centered practice, although they also revealed an awareness of not being confident in dealing with transgender patients. Finally, they recognized the need for training to improve their knowledge. Osteopathic person-centered care principles prove a valuable tool for practitioners to self-reflect on their compliance with an inclusive practice.

The themes will be depicted hereafter, supported by the most exemplificative participants’ quotations paired with the related identification code. These have been translated into English for clear understanding of this paper by non-Italian readers.

### 3.2. Microaggressions

Through verbal and non-verbal communication, nearly every participant demonstrated perpetuations of unconscious microaggressions based on cisnormativity, deadnaming and misgendering, improper language, and, finally, the idea of transgender people only being binary and medicalizing their experience.

Cisnormativity was expressed in its every meaning, thus showing a probably unconscious interiorized discrimination, which implies that being cisgender is “normal”, therefore pathologizing transgender people, talking about “such a condition” (O2), and excluding non-binary identities (Table 3).

**Table 3:** Cisnormativity.

*“well in the case for a male patient, if he had prostate surgery, one should ask for erection disorder, complaints about a series of secondary effects er… instead with a female patient after menopause or hysterectomy, an induced menopause indeed, one should ask if there are effects, consequences indeed within the sexual sphere” (O2)*
*“such a condition” (O2)*
*“a patient like that” (O5)*
*“I acknowledge the gender they appear as, and that’s more than enough for me” (O9)*
*“seeing is enough. I mean, if the patient’s appearance is plainly feminine, I’ll obviously use feminine pronouns, whilst if the patient appears as clearly masculine er… I use masculine pronouns” (O10)*

Deadnaming and misgendering are discriminatory behaviors that deny the gender affirmation of transgender people. It is important to remember that the gender identity of a person does not have to correspond with their gender expression as perceived by others, and that deadnaming can trigger gender dysphoria. Therefore, practitioners should never justify themselves with “it can happen, can’t it?”, as O5 tried to excuse themself. The deadname should be treated with the utmost confidentiality if the patient’s legal name still corresponds to their deadname and the practitioner needs it for reasons such as invoicing (Table 4).

**Table 4:** Deadnaming and misgendering.

*“It happened to me, and as soon as I said it, I understood it wasn’t… well, that the other name was preferable and so… I apologized, and I’ve used the other name, I mean, it can happen, can’t it?” (O5)*
*“The first case was a transgender Brazilian man” [talking about a transgender woman] (O10)*

Improper use of specific terminology; for instance, saying *“a person of this gender” (O5)*, as well as reification, i.e., *“the transgenders” (O1)*, shows little or approximate knowledge, leading to awkwardness in the relationship between patient and practitioner (Table 5).

**Table 5:** Improper language and reification.

*“MTF or FTM” (O1)*
*“the transgenders” (O1)*
*“is a gender” (O1)*
*“Let’s say they still had their original identity” (O2)*
*“not only gender” (O2)*
*“of the transgender situation” (O2)*
*“various choices of different gender” (O5)*
*“a person that has migrated their gender” (O5)*
*“this type of er… way of living” (O5)*
*“theme of transgenderism” (O5)*
*“someone not transgender” (O5)*
*“a person of this gender” (O5)*
*“this type of gender” (O5)*
*“this gender difference” (O6)*
*“transaction” (O6)*
*“the part related to the aspect of sex change” (O8)*

Even the most attentive participants showed a reductive idea of what being transgender means, understanding it only within the gender binarism and medicalization of transgender people, as exemplified by the idea of *“a different bodily component, because the body is in transition, and thus we find stiffness or anyhow, er, masses not corresponding to the patient’s biological origin, and so maybe the first time we deal with this body duality” (O1)*. Therefore, this binary concept is harmful and invisibilizes non-binary transgender people and those who do not undertake medical steps to reduce their gender dysphoria (Table 6).

**Table 6:** The idea of transgender people only as binary and medicalized.

*“waiting for how he or she defines himself or herself, if male or rather… and then adjusting and sticking to it, there!” (O1)*
*“a different bodily component, because the body is in transition, and thus we find stiffness or anyhow, er, masses not corresponding to the patient biologic origin, and so maybe the first times we deal with this body duality” (O1)*
*“during the case history, however, things come out when I ask for medication use, surgical history, and so certain things come out” (O7)*
*“when the definitive choice is made, and the process of physical change is undertaken” (O9)*
*“knowing the gender identity, therefore the possible absence or presence of hormonal information” (O10)*

### 3.3. Acceptance and Non-Judgement

Acceptance of people in a non-judgmental way was another emergent theme and is possible thanks to three fundamental elements: attention to names and pronouns, practitioner’s neutrality, and empathy. Practitioners should be aware of subconscious gender-related and sex biases, avoid distinguishing roles according to people’s sex or gender, and avoid discrimination from purported gender social roles.

Paying proper attention to name and pronouns is fundamental while welcoming transgender people, and the most common strategy is listening to a patient’s story *“I hardly ask questions but listen a lot. Therefore, letting one talk, then… let’s say, the better way to relate to each other will emerge […] if I wouldn’t be able to er use this type of strategy, I’d frankly and honestly ask how they prefer to be called” (O10)*. Avoiding grievous errors such as deadnaming and misgendering subsequently makes the person feel accepted and safe, as everyone should feel within a healthcare setting. This is illustrated by O7: *“I reckon it is needed to put the patient at ease”*. Even though participants expressed the importance of this, they only considered transgender people as binary and medicalized, e.g., *“as they arrived and introduced themselves with a feminine or masculine name, I adjust my way of talking based on what they say” (O7)* (Table 7).

**Table 7:** Name and pronouns attention.

*“Yes. Because maybe I don’t always have the intimacy of… asking it” [referring to the possibility of extrapolating the patient’s preferred pronouns out of their story] (O4)*
*“I really ask how they want to be called. […] It is the most natural way for empathic communication. […] inclusivity is everything” (O6)*
*“as they arrive and introduced themselves with a feminine or masculine name, I adjust my way of talking based on what they say” (O7)*
*[talking about the importance of knowing patients’ gender identity in order to use correct pronouns] “I reckon it is needed to put the patient at ease” (O7)*
*“since he told me “my name is [masculine name]” I’ve always called him so” (O8)*
*“yes, absolutely. Actually, I daresay, it’s the only way to know how patients consider themselves, apart from everything, therefore yes” [referring to the possibility of extrapolating patient’s preferred pronouns out of their story] (O9)*
*“if the patient is feeling in a specific… way, I respect his feeling” (O10)*
*“I hardly ask questions but listen a lot. Therefore, letting one talk, then… let’s say, the better way to relate to each other will emerge […] if I wouldn’t be able to er use this type of strategy, I’d frankly and honestly ask how they prefer to be called” (O10)*

Practitioner neutrality, i.e., *“entering into the person’s context without judging, but actually only evaluating” (O6)*, is effective because it improves the patient–practitioner encounter with people from every background, not only transgender people, as O7 explained: *“talking about practitioner’s neutrality, besides the person in front of you, I reckon it would really help, in the broadest spectrum”* (Table 8).

Alternatively, empathy is presented as one of the central elements of osteopathy. O8 illustrates this as “*in my opinion, the fundamental thing is, independently from the subject of transgender people […], in our job what matters a lot is empathy for sure*”. This allows for a non-judgmental approach and welcoming whoever the patient is (Table 9).

**Table 8:** Practitioner’s neutrality.

*“one must try to behave […] without appearing to judge or even giving a hint of judgment” (O2)*
*“a comfortable environment, a non-judgmental environment er… can result in an er… happier expression and a more peaceful life of the transgender person” (O4)*
*“I’ve developed a communication that always tries to be neutral” (O6)*
*“entering into the person’s context without judging, but actually only evaluating” (O6)*
*“try to be the most neutral possible, but with every patient […] Therefore the word partner is a word that, when talking about it, I often use because anyhow I don’t know who is in front of me” (O7)*
*“talking about practitioner’s neutrality, besides the person in front of you, I reckon it would really help, in the broadest spectrum” (O7)*

**Table 9:** Empathy.

*“in my way of being, er, towards the patient there is anyhow, first of all, a person […] thus one must behave, yes, in an empathic and non-judgmental perspective” (O2)*
*“I think that in our job a high amount of empathy is needed” (O4)*
*“trying as much as possible to enter em-pathos, that is feeling and perceiving passions, and this can be made only and exclusively without judgment” (O6)*
*“the importance of empathy which is a huge enabler [of the therapeutic process]” (O6)*
*“in my opinion, the fundamental thing is, independently from the subject of transgender people […], in our job what matters a lot is empathy for sure” (O8)*

### 3.4. Person-Centered Treatment

The participants reported a characteristic element of osteopathic person-centered care, focusing on the person rather than on the disease. This could be a reason for the lack of practice protocols, because every treatment is individualized to the patient’s needs and uniqueness. The interviews showed how this does not differ in transgender patient treatment or when multidisciplinary care is needed.

All the participants emphasized how the treatment of transgender people does not hinge on being transgender. Therefore, this does not differ from treatments for cisgender people in its person-centered approach, as O9 clarified: *“We take care of the person, not the patient, not their choices […]. In my opinion, that is the focus, that’s important for everyone, therefore the fact that one could be a transgender person subsequently loses significance […] it’s not anymore that characteristic that interests you, but the fact that is a person addressing to you for needs and necessities”*. This might be explained due to practitioners constantly modulating both approaches and palpations from one patient to another, based on the patient’s needs. The patient is considered a human being, and therefore equal to others, but concurrently unique (Table 10).

**Table 10:** No differences in the treatment compared with cisgender people.

*“it can be said that all patients are absolutely unique and different, but are equal in the way one tries to understand them within their own […] therapeutic environment” (O5)*
*“a person-centered approach and any discrimination that could distort the good treatment outcome should not exist […] these treatments are shaped […] on the human being (i.e., physical, cognitive, social, emotional, and existential domains)” (O6)*
*“we welcome and treat any person entering into our studio, beyond the fact of a patient’s gender, sex, age, social class… we treat people” (O7)*
*“I answer you yes for the [treatment] modulation, but not only with him, that is to say, I treat no patient in the same way, with the same intensity, I adapt to the requests of the tissues I’m handling” (O8)*
*“We take care of the person, not the patient, not their choices […]. In my opinion, that is the focus, that’s important for everyone, therefore the fact that one could be a transgender person subsequently loses significance […] it’s not anymore that characteristic that interests you, but the fact that is a person addressing to you for needs and necessities” (O9)*
*“In my mind, inclusivity is also considered as normal, which for many people is not. Therefore, giving less importance… or rather, attention is fair, but I pay attention to anyone […] paying attention to LGBT people, in my opinion, is part of the framework of… paying attention to people” (O10)*

A multidisciplinary approach also follows person-centered assistance tenets: other practitioners’ support is always sought if patients would need it, independently of their gender identity, exemplified by *“I reckon it highly depends on the actual person’s requirements, for the reason they are coming to us. […] There can obviously be some conditions, other than the context of being transgender, that can require a multidisciplinary collaboration regardless, and thus, I mean, in my opinion also this is evaluated depending on the person and not on this characteristic” (O9)* (Table 11).

**Table 11:** Multidisciplinarity.

*“a correct [patient] takeover depending on the person’s necessities is fundamental […] it is a pyramidal approach which has to be informed by the actual person’s needs” (O6)*
*“multidisciplinary collaboration […] I consider it a bit fundamental for everyone” (O7)*
*“I reckon it highly depends on the actual person’s requirements, for the reason they are coming to us. […] There can obviously be some conditions, other than the context of being transgender, that can require a multidisciplinary collaboration regardless, and thus, I mean, in my opinion also this is evaluated depending on the person and not on this characteristic” (O9)*
*[talking about multidisciplinarity] “it won’t be different from the subject also arising from other patients’ backgrounds. That is, it doesn’t add any specific requirements because the patient is a transgender person” (O10)*

### 3.5. Education Implementation

The last theme which emerged was the need for adequate education. Indeed, more than half of the participants expressed awkwardness or difficulty in communication. Practitioners can acquire interpersonal competencies during their education as well as personal and continuous professional development.

Regarding the difficulties reported, these expressly concern the social side: the uncertainty arising from not exactly knowing how to relate to this group of patients, the problem of adapting language, and the awareness of delving into little-known themes where one can supposedly easily make mistakes, as O1 recalled: *“I remember the first times […] in the waiting for the treatment to start I had a thousand questions in my mind […] I asked myself “how to approach, what do I do, what do I say?””* (Table 12).

Some participants addressed these difficulties through research and active development, a fundamental requirement for an informed practitioner, as O4 stated, *“it’s anyhow a really modern topic, that updates itself, people have to keep themselves up to date […] I think that a good ability of any good therapist is to be always up to date, also about this category of social situations”* (Table 13).

**Table 12:** Difficulty and awkwardness expressed by the practitioner.

*[talking about the awkwardness at the patient’s coming out] “I remember the first times […] in the waiting for the treatment to start I had a thousand questions in my mind […] I asked myself “how to approach, what do I do, what do I say?”” (O1)*
*“my difficulty, I mean, then at the end, er, it’s kind of… […] difficulty of, of being able to enter such an intimate sphere…” (O2)*
*“at first maybe there was some difficulty to have… to adapt the language” (O2)*
*“in general, I try to treat the subject with great tact, because I don’t consider myself an expert who… that is, I know I may be sailing in a territory I don’t know very well” (O4)*
*“it happens that culturally you stumble in specific modalities or because such verbs, nouns, phrases are still to be found, that haven’t come yet into use to broach the subject” (O5)*
*[talking about the awkwardness at the patient’s coming out] “at first, yes. […] Then to the reality of the facts, when I saw this person physically in the studio, the thing changed at once, […] and then what could have been all a house [of cards] I’d basically created in my mind was practically demolished within five minutes of conversation” (O8)*

**Table 13:** Personal development.

*“it’s anyhow a really modern topic, that updates itself, people have to keep themselves up to date […] I think that a good ability of any good therapist is to be always up to date, also about this category of social situations” (O4)*
*“It gave me perhaps the desire to go deeper into a topic that before instead, but not for anything else, not for lack of desire or other, but just for lack of direct relationship I wouldn’t do for sure, so it certainly gave me that little something extra” (O8).*

All participants recognized the necessity of an osteopathic curriculum that integrates thorough knowledge of social, individual, and biological diversity. This is illustrated in, for example, *“I reckon that a more in-depth work should be done not only concerning transgender people, but including all those categories that are now still being identified as different categories” (O8)*. Indeed, education shapes our belief system, as O10 pointed out *“Personally maybe not, because anyhow I’ve got a certain type of background […] if I were, you know, an average student […] therefore very sensitive to the contents delivered by the school, absolutely yes. I mean, this is the point of school, because, anyhow, it creates a cultural construction, a thought structure”* (Table 14).

**Table 14:** Need to implement the educational program.

*“I’d say yes. But maybe in the form of Medical Humanities […] for a healthcare practitioner it is fundamental to acquire in equal measure the ethical relational contribution, you know, so a subject precisely including the relationship with social realities” (O1)*
*“if a person hears a lot about it but maybe hasn’t any type of… prejudice or problem, as it should be, but maybe one lacks certain information that can effectively make a person prepared on the subject” (O4)*
*“It’s important that those who don’t know this phenomenon or who have their own difficulties, through improving their knowledge, can improve their ability to make contact […] I reckon that also from the biomedical point of view, more precisely biological, an in-depth knowledge is needed, not only from the psychosocial point of view” (O5)*
*“if we succeed within the healthcare practitioners training […] to underline Hippocratic concepts, er, and so of non-distinction, of non-judgement […] it’s plain that everything would be already included […] Even if […] educating with dedicated time for some topics would simplify therapeutic processes […] I’m pretty sure that uhm that differences brought from social superstructures would almost be evened out because we don’t care what you do, but who you are” (O6)*
*“I reckon that a more in-depth work should be done not only concerning transgender people, but including all those categories that are now still being identified as different categories” (O8)*
*“it certainly should be put within the global of all the bioethical implications of healthcare professions, when dealing of the field of professional ethics […] also this one should be done” (O9)*
*“Personally, maybe not, because anyhow I’ve got a certain type of background […] if I were, you know, an average student […] therefore very sensitive to the contents delivered by the school, absolutely yes. I mean, this is the point of school, because, anyhow, it creates a cultural construction, a thought structure” (O10)*

## 4. Discussion

The findings in this study demonstrate the existence of microaggression phenomena in osteopathic practice, which reflect cisnormativity within society [6,11,13,14], and particularly the Italian socio-cultural context [43]. These behaviors lead to the stigmatization of transgender people [7,14,21] and result in the denial of gender affirmation through deadnaming and misgendering [7,13], provoking discomfort for transgender people dealing with healthcare practitioners [44]. This occurs mainly with non-binary transgender people, who constantly see their identity erased by incessant misgendering [9,44]. This inability to fathom non-binary identity [9] is demonstrated by the participants’ belief that all transgender people are binary and medicalized. In contrast, many transgender people have a non-binary identity, show a non-conforming gender expression, or do not wish to undertake gender transition [6,9,14,44].

This research also identified deadnaming and misgendering phenomena, in addition to the use of potentially nocebic (i.e., harmful, the opposite of placebic) language and communication, which display inadequate or superficial knowledge of transgender people’s needs [6]. Several points, in particular, were observed: confusion in the use of basic terminology [6]; language biologic objectification, and thus the use of terms such as FTM (female-to-male) instead of AFAB (assigned female at birth) which assume physical characteristics and binary gender identity [9,11]; reification [45] of transgender people as part of the stigmatizing Italian social climate [43].

The language chosen by the practitioner has extraordinary positive and negative effects on the patient [1]; therefore, a gender-affirming discourse is more than fundamental [46]. The participants who declared that they pay particular attention to names and pronouns also recognized this importance. It shows respect and defines the positivity of the experience, contributing to the patient non-avoidance of future appointments [6,46,47].

Furthermore, participants highlighted language importance, empathy, and practitioner’s neutrality as crucial in welcoming the patient. This finding conforms within the literature as empathy and is described as part of the interpersonal relationship between patient and practitioner, eliciting a placebo effect operating on the patient’s affective state [1,48]. Moreover, empathy is conveyed through touch, facilitating the establishment of a therapeutic alliance [49]. Osteopaths’ typical neutrality is linked to a positive first impression and an inclusive practice that communicates safety and makes the patient feel welcome in the care environment [6,14,48].

The content analysis also showed how treatments are person-centered [2], thus focusing on effective interpersonal relationships [50]. This approach should not differ in the case of a transgender patient. Nevertheless, a gentle touch in osteopathy is a requisite for good therapeutic relationships and the effectiveness of treatments [1,49]. However, the chance of interiorized discrimination, herein highlighted by the microaggression phenomenon, could be conveyed through touch, overcoming the verbal component in which discrimination could be masked by the practitioner [49,51].

From the interviews, it has also emerged that most participants are not able to deal with transgender patients due to the lack of appropriate education and a cisnormative society [6,11].

During the interviews, the participants went through a self-reflective process identifying their lack of competencies while describing their experience with transgender patients. The osteopathic person-centered principles could represent a practitioners’ guide for self-reflection, to mirror and drive the clinical reasoning, and recognize the needs of the patient as well as those of the practitioner. This could, for example, be used to highlight gaps in knowledge and select appropriate continuing professional development programs. The osteopathic tenets have recently been proposed as a framework to approach sexual orientation and gender identity disclosure more appropriately in the healthcare environment [28]. Osteopathic practitioners are encouraged to reflect and act considering the person not just as a body, or only considering changes in the body, but rather referring to a dynamic unity that involves the mind and spirit [28]. We know that reflection underpins all osteopathic practices and continuing professional development [52]. Reflective practices may help prevent error and bias and facilitate practitioners in improving patient care. Moreover, it should improve practitioner wellness and job satisfaction [52].

To address this gap, a culturally safe environment must be created [6] to avoid stigmatizing patients at interpersonal and structural levels [14,21]. Hence, education implementation is required to avoid discriminatory behaviors and the patient educating the practitioner about their needs [6,11,46]. A gender-affirming environment is indispensable to support transgender patients’ health [46]; therefore, creating awareness about transgender people’s needs is essential [9,11,46]. The aforementioned cultural competency is crucial for a person-centered approach [47]. Cultural competency can be accomplished by introducing new curricular activities involving the transgender community to be more efficacious [6,46,47]. Clinic-based learning simulations with standardized patients (i.e., transgender actors or cisgender actors who have studied in close contact with transgender people) can effectively educate students and tackle discrimination [21]. Hollenbach and colleagues published a specific document for further proposals to implement education [53]. Other resources include the Standard of Care defined by the World Professional Association for Transgender Health (WPATH) [54], the guide for LGBTQIA+ patients care outlined by the Joint Commission [55], and the conferences providing course credits proposed by the Gender Odyssey [56].

Indeed, there is currently a developing interest from different research groups involved in research in healthcare experiences for sexual and gender minority people. Various researchers have focused on these LGBT populations using a variety of different types of studies, such as audits [57], observational studies [58], qualitative research [59], mixed-methods designs [60], and a current protocol for a systematic review [61], to monitor the improvement in health, well-being, and healthcare experiences for sexual and gender minority people. This research is likely to contribute to the better understanding of any issues as well as further opportunities to address them.

Nevertheless, we know from recent research findings that the failure to gather data on LGBTQIA+ identification in healthcare (including osteopathic) practice and education is part of a more significant issue that restricts addressing LGBTQIA+ health inequities [57]. For example, a recently published survey revealed that American medical education institutions undervalued sexual orientation and gender identity in demographic collection procedures, with osteopathic programs reporting less inclusive best practices in several areas than allopathic programs. As part of a comprehensive effort to address sexual and gender minority health inequalities, healthcare education institutions must alter their methods to collect sexual orientation and gender identity demographics [62].

We acknowledge some limitations, the first of which is that the study was limited to Italian osteopaths. Second, the results may not be fully generalizable to other countries or circumstances, nor can they be used to represent the entire osteopathic profession; however, future studies with participants from other countries could provide data for an overall picture. Additionally, because some of the participants had only interacted with one transgender patient, to obtain a broader view of the phenomenon, further studies should be conducted to explore the perspectives of osteopathic physicians who have had extensive clinical experience with sexual and gender minority people.

This study addresses a gap in the scientific literature within osteopathic care. To the best of our knowledge, this content analysis is the first qualitative study on this topic in osteopathy. Hence, future studies, including clinical audits and cross-sectional observational studies with representative samples, should focus on transgender patients to understand their perspective on those aspects needing improvement to ensure a fully inclusive osteopathic practice. Students’ attitudes also need to be investigated to understand how to train new practitioners most effectively, providing them with the necessary skills to deal with this social reality.

## 5. Conclusions

This qualitative study investigated Italian cisgender osteopaths’ attitudes in the care of transgender people and their desire to embrace and apply the core osteopathic tenets, regardless of the patient’s gender identity. Notwithstanding, even though the Italian osteopaths studied may be more inclusive than other healthcare practitioners, they are still affected by the social climate and informational erasure. It is vitally important to train culturally competent practitioners who can recognize and avoid microaggressions and thus challenge cisnormativity, making osteopathic practice inclusive towards transgender people and substantially contributing to ensuring their good psychophysical health.

## Figures and Tables

**Table 1:** Interview saturation process.

Interview number	1	2	3	4	5	6	7	8	9	10	11	12	13	14	15	16	17	18
Base themes	2	2	1	1	3	0	0	0	0	2	0	0	0	0	0	0	0	0
New themes in run				6		3	0	0	0	2	2	0	0	0	0	0	0	0
% change over base (threshold of ≤5%)						50%	0%	0%	0%	33.3%	33.3%	0%	0%	0%	0%	0%	0%	0%

**Table 2:** Overarching theme, themes, and categories.

Overarching Theme *	
Principles of person-centered osteopathic care as a tool for monitoring inclusive practice	
Themes **	Categories ***
Microaggressions	Cisnormativity
	Deadnaming and misgendering
	Improper language and reification
	The idea of transgender people only as binary and medicalized
Acceptance and non-judgment	Name and pronoun attention
	Practitioner’s neutrality
	Empathy
Person-centered treatment	No differences in the treatment compared with cisgender people
	Multidisciplinarity
Education implementation	Difficulty and awkwardness expressed by the practitioner
	Personal update
	Need to implement the educational program

* Overarching themes tend to organize and structure an analysis; they capture an idea underpinning a number of themes but are rarely analyzed themselves. ** A category is a collection of similar data sorted by the same meaning, and this arrangement enables the researchers to identify and describe the characteristics of a theme. *** A theme is a meaningful “essence” that runs through the data.

## Data Availability

The Italian version of the original data has been translated to be understandable for non-Italian readers (see Table 3, Table 4, Table 5, Table 6, Table 7, Table 8, Table 9, Table 10, Table 11, Table 12, Table 13 and Table 14). The Italian version of the questions and the prompts used for the semi-structured interviews and the participants’ quotes can be requested from the corresponding author. The data presented in this study are available on request from the corresponding author.

## Appendix A

**Table A1:** Abbreviation list and glossary.

Terms	Definition
Binary transgender person	A transgender person who identifies with the opposite gender from that which was assigned at birth, i.e., men who were assigned female at birth (AFAB) and women who were assigned male at birth (AMAB) [5,8,9]
Cisgender	People who identify themselves with the gender assigned at birth.
Cisgenderism	Discrimination based on different gender identities, and therefore, on being transgender [22].
Cisnormativity	The assumption that everyone is cisgender, that gender is only binary, and the pathologizing of transgender people [11,13,14].
Deadname	The former, “dead”, name of a transgender person, usually given by their parents. The deadname often triggers dysphoria. For this reason, transgender people mostly choose a new suitable name, their “chosen name”. The deadname must not be asked for, and if it is known, for whatever reason, it must not be made public without the explicit consent of the person.
Deadnaming	The act of using the deadname is a serious form of discrimination, and it is still harmful even if the person being named is not present.
Gender affirmation	The way cis- and transgender people express their gender through clothing, hairstyle, etc. Gender expression may differ from that expected from the dominant culture gender stereotypes, but is still valid and authentic.
Gender transition	A process characterized by having different aspects (social, medical, etc.) and is unique to each individual. It might include changing their name, gender marker, clothing style, speech training, make-up, endocrine therapy and/or surgery, and others, aiming to reduce the different causes for gender dysphoria and affirming the transgender person’s gender identity.
Heterosexism	Discrimination based on a sexual orientation other than heterosexuality [22].
Invisibilize	The act of making a minority group invisible—ostracizing.
Intersex people	People born with several biological sex characteristics not conforming with male–female binarism. These characteristics can also develop naturally during puberty.
LGBTQIA+	Lesbian, gay, bisexual, transgender, queer/questioning, intersex, asexual/aromantic/agender, and all other nuances within the community; the + is used to shorten the full initialism LGBTQIAPK, in which the missing letters are for polyamorous/pansexual and kink.
LGB	Lesbian, gay, and bisexual.
Microaggressions	Behaviors, affirmations, and environmental messages that convey, even subconsciously, hostile messages about ethnicity, gender, sexual orientation, and religion [4].
Misgendering	Using salutations and pronouns not matching with the gender identity of the concerned person. */Ə/y/u can be used instead of vowels to correctly refer to a non-binary transgender person.
Non-binary transgender person	A transgender person whose gender identity goes beyond female–male gender binarism and thus identifies as non-binary, genderfluid, queer, or agender [5,8,9].
Reification	The tendency to depersonalize an individual, reifying them to a mere object. It is the opposite of “person-first language”, a language showing respect for human uniqueness. Reifying transgender people means defining them out of existence that involves far more than biology. Using an adjective as a noun, i.e., objectivization, erases the complexity of the person, who is identified only with that specific characteristic [45]. For example, saying “transgenders” instead of “transgender people”.
Transgender	An umbrella term that includes every person with a different gender identity from their gender assigned at birth, which includes both binary and non-binary transgender people. Some transgender people could feel the need to transition (see “gender transition” above). [5,8,9,11].

## Appendix B

### Appendix B.1. Interview I

#### Appendix B.1.1. Relationship between Patient and Practitioner

‑ Have you ever found yourself unprepared after a patient’s coming out? If yes, what have you felt and how have you dealt with the situation? Have you researched afterwards to fill your gaps?
‑ When talking to the patient, are you careful to use inclusive and gender-affirming language? Do you always ask for which pronouns to use? If it happened that you misused the pronoun, did you promptly apologize?
‑ Have you been careful to use neutral language? For instance, “partner” instead of “boyfriend” or “girlfriend”?
‑ Have you ever mistaken and used the deadname while talking with the patient? Have you ever shown curiosity for the patient’s deadname? Do you know that even simple curiosity is inappropriate and puts the patient in an uncomfortable position?
‑ If the patient hasn’t already changed their official documents, how do you behave when issuing invoices or other documents in which the legal name is needed? What do you do to make the patient feel equally accepted?
‑ Have you ever noticed to have perpetuated other forms of cisgenderism and transphobia, either verbal or non-verbal? For example, asking inconsistent queries with the reason for the consultation or conducting narrow-minded behaviors. If so, how did you make amends?
‑ Have you changed your palpation to meet the patient’s needs? If yes, how?
‑ Are you careful to monitor for possible unwanted microaggressions? What do you do to avoid them?

#### Appendix B.1.2. Case History Taking

‑ Collecting patient data, do you only fill in the sex corresponding to the patient’s gender expression, or do you precisely ask for gender identity and sexual orientation? If so, how do you ask for it, with pre-established options or an open question? If not, are you considering implementing this aspect in your case history? Do you find it important for patient care?
‑ What other data do you collect? Gender assigned at birth? The presence of organs and undertaken surgeries? Do you use neutral language in doing so?

#### Appendix B.1.3. Clinic Environment

‑ What do you do to make your waiting room more welcoming? Do you place LGBT flyers, newspapers, or posters?
‑ If you have a welcome desk, have you been careful that they are LGBT-friendly before hiring them? Have you recommended them to avoid transgender people’s deadnames, even if not yet changed on official documents?
‑ In your studio or clinic, are the bathrooms gendered or is there only one bathroom? Do you know that gendered bathrooms are uncomfortable for transgender people, especially non-binary ones?

### Appendix B.2. Interview II

#### Appendix B.2.1. Skills

‑ To what do you attribute the ability to treat without discriminating transgender patients? For instance, considering the person before the patient, or the capacity to empathize towards patients, or considering all patients equal?
‑ Do you prefer to listen to the patient’s tale instead of asking questions? Do you think this could be a strategy to extrapolate and use the person’s preferred pronoun?

#### Appendix B.2.2. Multidisciplinarity

‑ Is multidisciplinary collaboration important to you in the care of a transgender person? Which other practitioners do you consider necessary?
‑ Do you reckon the psychological support as important for a transgender patient? Explain your answer.

#### Appendix B.2.3. To Be Implemented

‑ Do you think that introducing social and medical aspects about transgender people within osteopathic university training is necessary? Do you reckon that you would have felt prepared to face this patient category, having studied such matters?

## Appendix C

**Table A2:** Demographic data.

Participants’ ID n.	Age	No. of Years in the Profession	Religious Affiliation	Sexual Orientation	Gender Identity
O1	47	19	non-practicing Catholic	heterosexual	cisgender
O2	56	33	none	heterosexual	cisgender
O3 *	45	10	atheism	heterosexual	cisgender
O4	28	4	Catholic	heterosexual	cisgender
O5	58	22	none	heterosexual	cisgender
O6	42	12	atheism	heterosexual	cisgender
O7	40	4	non-practicing Catholic	heterosexual	cisgender
O8	33	8	non-practicing Catholic	heterosexual	cisgender
O9	43	20	none	heterosexual	cisgender
O10	60	20	none	heterosexual	cisgender

* Drop out.
